# Supplementary material for: Strategies to investigate and mitigate collider bias in genetic and Mendelian randomisation studies of disease progression
Source: PLoS Genet. 2023 Feb 23;19(2):e1010596. doi: 10.1371/journal.pgen.1010596 (PMC9949638; doi:10.1371/journal.pgen.1010596)
Supplement: S1 Text — Fig A in S1 Text. Directed acyclic graph illustrating potential pleiotropic pathways from LDL-C and HDL-C to CHD mortality. DAG illustrating potential pleiotropic pathways from LDL-C and HDL-C to CHD mortality. Solid black lines indicate assumed causality. Absent solid lines indicate assumed lack of causality. Dashed red lines indicate induced associations. Absent dashed red lines indicate lack of induced associations. Boxes indicate a variable that has been conditioned on. Table A in S1 Text. One-sample MR estimates of the effect of lipid traits on CHD onset in UK Biobank (N = 337,288 eligible). Estimates are among adults of European ancestry and are adjusted for age, sex, and the first ten genetic principal components. Models are two-stage prediction substitution regression models with bootstrapped standard errors (100 replications). Table B in S1 Text. One-sample MR estimates of the total effect of lipid traits on statin use in UK Biobank. Estimates are among adults of European ancestry and are adjusted for age, sex, and the first ten genetic principal components. Models are two-stage prediction substitution regression models with bootstrapped standard errors (100 replications). (DOCX) [file pgen.1010596.s001.docx]

# **S1 Text: Supplementary Information**

## Methods

We used individual-level data from the UK Biobank (UKB), a prospective cohort study in which 502,549 adults aged 38-69 years were recruited between 2006-2010 via 22 assessment centres across England, Wales, and Scotland (1) (~5% response rate (2, 3)). The study design, participants, and quality control (QC) are detailed elsewhere (4). Participants provided written informed consent. Ethical approval was obtained from the Northwest Multi-centre Research Ethics Committee (11/NW/0382). Data were accessed via application number 16391.

Nearly all participants provided blood samples at the 2006-2010 clinic for genotyping and biochemistry analyses. Genotype was measured from serum samples using a genome-wide array (UK Biobank Axiom Array) with imputation to the Haplotype Reference Consortium panel. Pre-imputation QC, phasing, and imputation are described elsewhere (5). Our analyses were restricted to autosomal variants using graded filtering with varying imputation quality for different allele frequency ranges (6). 814 individuals with a mismatch between genetic and reported sex, and with sex-chromosome aneuploidy, were excluded. We further restricted to individuals of ‘European’ ancestry as defined by k-means clustering using the first 4 principal components provided by UKB (6). We included the largest cluster from this analysis (n=464,708 eligible for subsequent analyses).

We constructed genetic risk scores (GRS) for LDL-C and HDL-C from a published genome-wide association study (GWAS) that excludes UKB (7), including 33 and 44 single nucleotide polymorphisms (SNPs), respectively (8). For the purposes of multivariable MR adjustments for HDL-C, we additionally constructed a GRS for triglycerides based on 16 SNPs (7) and for apolipoprotein B based on 14 SNPs from targeted metabolomics (nuclear magnetic resonance spectroscopy) that also excludes UKB (8). GRSs were made using PLINK 2.0, with GWAS effect alleles and betas as weightings. Standard scoring was applied by multiplying the effect allele count (or probabilities if imputed) at each SNP (values 0, 1, or 2) by its weighting, summing these, and dividing by the total number of SNPs used. The score therefore reflects the average per-SNP effect on the exposure. GRSs and their respective exposure traits were each standardised into z-score (SD) units for analyses.

CHD history was defined using inpatient ICD-10 codes as having developed before the date of baseline clinic assessment a primary or secondary diagnosis of CHD (I20, I200, I201, I208, I209, I21, I210, I211, I212, I213, I214, I219, I22, I220, I221, I228, I229, I23, I230, I231, I232, I233, I234, I235, I236, I238, I24, I240, I241, I248, I249, I251, I252, I255, I256, I258, I259). Mortality with CHD as a primary or secondary cause was defined using the same ICD-10 codes, with a median (range) follow up time of 11.2 (0.01 - 14.1) years. Among 357,840 participants eligible for current analyses (i.e., who had data on either lipid exposure and its GRS, plus age, sex, genetic PCs, CHD history status, CHD mortality status, and weightings for IPW adjustments), 20,552 (5.7%) had a pre-baseline history of CHD, and 2,625 (0.7%) later had a recorded death from CHD. Of those who died of CHD, 44.5% had a recorded CHD history, whilst 55.5% did not.

To verify expectations of the causality of lipids for CHD incidence, we estimated the effects of LDL-C and HDL-C on the risk of CHD onset, with CHD onset defined using inpatient ICD-10 codes as having developed after the date of baseline exposure assessment a primary or secondary diagnosis of CHD (coded as above), among adults without those diagnosis codes at the time of baseline exposure assessment. Two-stage least squares (predictor substitution) regression models were used for this, where in a first-stage linear model, e.g., LDL-C is regressed on the GRS for LDL-C, plus age, sex, and the first ten genetic principal components (GPCs). The predicted values from that model were then entered into a logistic model as an exposure (plus age, sex, GPCs) with CHD onset as the outcome.

IPW was performed by weighting each regression stage (first stage linear and second stage logistic) for the inverse probability of having had CHD (and survived) before the date of baseline clinic assessment based on predicted values from a separate logistic model of CHD case status regressed on sex, age, highest educational qualification, smoking status, alcohol status, body mass index, waist-to-hip ratio, and relative grip strength (maximum grip divided by weight in kg). Weightings therefore took the form of ‘1 / predicted values’ for those with CHD history.

We estimated the prevalence of statin use based on medication codes for self-reported use of any of 13 drugs (atorvastatin, crestor, eptastatin, fluvastatin, lescol, lipitor, lipostat, pravastatin, rosuvastatin, simvador, simvastatin, zocor, zocor heart-pro) as defined in previous genetic analyses of UKB (9). We used this composite statin variable (yes/no) to estimate the effects of LDL-C and HDL-C on statin use using the same two-stage one-sample MR modelling approach described above with logistic regression as the second stage.

#

The two-stage models for LDL-C and HDL-C were each initially univariable. Each lipid was then analysed in multivariable models with mutual adjustment and with additional adjustment for triglycerides, given expectations of pleiotropy (apolipoprotein B is expected to be the predominant causal factor underpinning effects of LDL-C, but apolipoprotein B was not adjusted for as it is a feature of LDL particles and its adjustment would not be appropriate for LDL-C; the trio of LDL-C, HDL-C, and triglycerides enabled a consistent multivariable adjustment set across exposures) (10, 11). For example, first-stage multivariable models for HDL-C were adjusted for measured LDL-C, the GRS for LDL-C, measured triglycerides, the GRS for triglycerides, age, sex, and GPCs. The second-stage multivariable models for HDL-C were then adjusted for genetically-predicted LDL-C, genetically-predicted triglycerides, age, sex, and GPCs (for the purpose of these models, genetically-predicted triglycerides were based on multivariable adjustments for HDL-C and LDL-C, and the GRS for each, in first-stage models).

Results of these models for CHD incidence supported our expectations of causality, with LDL-C raising the odds of CHD onset, at 1.65 (95% CI = 1.43, 1.91) times higher (**Table A**); this attenuated but remained substantially elevated upon multivariable adjustment for HDL-C and triglycerides (OR = 1.23, 95% CI = 1.10, 1.38). In univariable models, higher HDL-C appeared to reduce the odds of CHD onset, at 0.86 (95% CI = 0.77, 0.96) times lower. Upon multivariable adjustment for LDL-C and triglycerides, however, this estimate attenuated towards the null, e.g., to an OR of 0.94 (95% CI = 0.86, 1.02) (**Table A**), suggesting that HDL-C does not substantially directly affect CHD onset.

**Fig A. Directed acyclic graph illustrating potential pleiotropic pathways from LDL-C and HDL-C to CHD mortality.**

DAG illustrating potential pleiotropic pathways from LDL-C and HDL-C to CHD mortality. Solid black lines indicate assumed causality. Absent solid lines indicate assumed lack of causality. Dashed red lines indicate induced associations. Absent dashed red lines indicate lack of induced associations. Boxes indicate a variable which has been conditioned on.

**A**

*
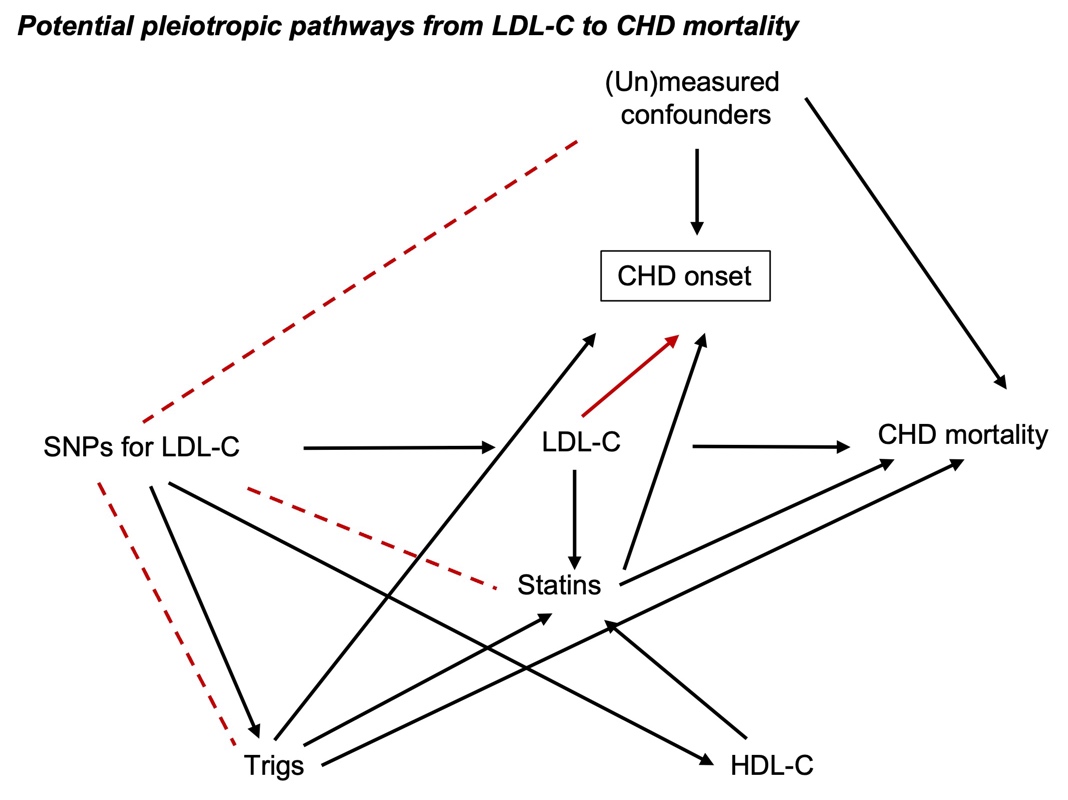

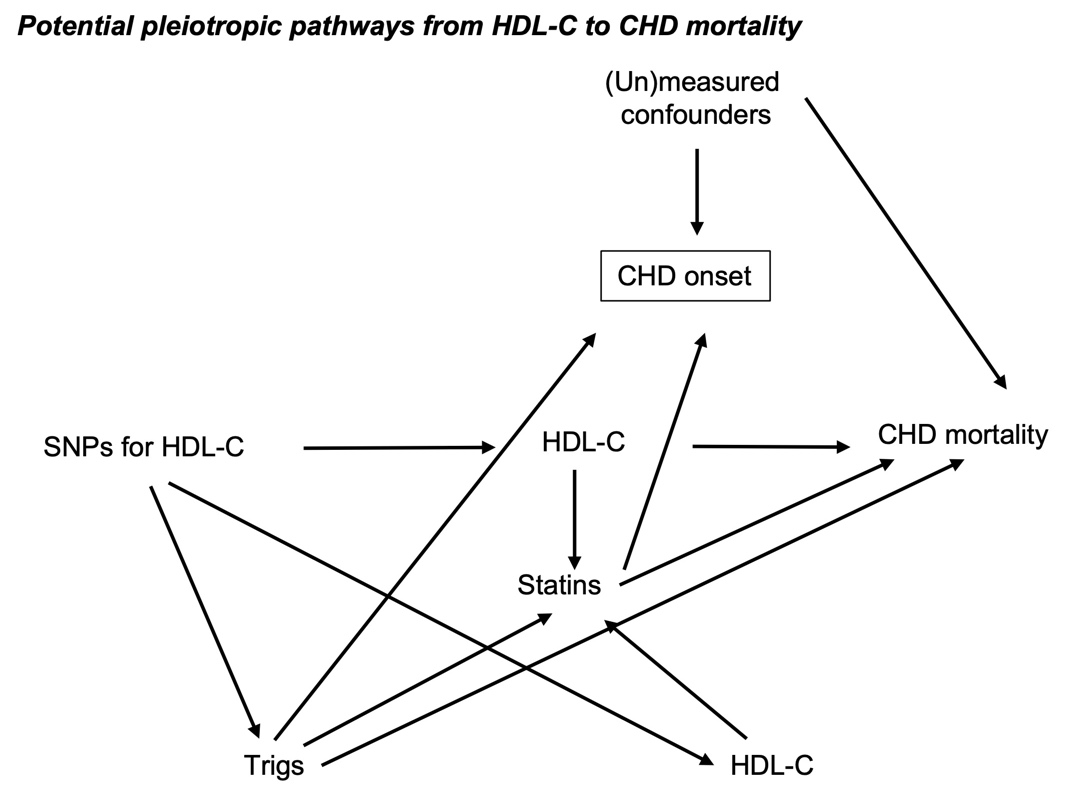
*

**B**

| **Table A** One-sample MR estimates of the effect of lipid traits on CHD onset in UK Biobank (N=337,288 eligible) | |
| --- | --- |
|  |  |
|  | **OR (95% CI) for CHD onset per SD higher lipid trait** |
| **Positive effect expected** |  |
| LDL-C | 1.65 (1.43, 1.91) |
| LDL-C, adj. for HDL-C & trig. | 1.23 (1.10, 1.38) |
|  |  |
| **Inverse effect expected** |  |
| HDL-C | 0.86 (0.77, 0.96) |
|  |  |
| **Null effect expected** |  |
| HDL-C, adj. for LDL-C & trig. | 0.94 (0.86, 1.02) |
|  |  |
| Estimates are among adults of European ancestry and are adjusted for age, sex, and the first ten genetic principal components. Models are two-stage prediction substitution regression models with bootstrapped standard errors (100 replications). | |

| **Table B** One-sample MR estimates of the total effect of lipid traits on statin use in UK Biobank | | |
| --- | --- | --- |
|  | |  |
|  | **OR (95% CI) for statin use per SD higher lipid trait** | |
|  |  |  |
|  | **Among adults overall**  N=357,840 eligible | **Among adults with CHD history**  N=20,552 eligible |
|  |  |  |
| LDL-C | 3.85 (3.59, 4.13) | 5.71 (3.62, 9.00) |
|  |  |  |
| HDL-C | 0.76 (0.73, 0.80) | 0.82 (0.71, 0.95) |
|  |  |  |
| Estimates are among adults of European ancestry and are adjusted for age, sex, and the first ten genetic principal components. Models are two-stage prediction substitution regression models with bootstrapped standard errors (100 replications). | | |

## References

1. Littlejohns TJ, Sudlow C, Allen NE, Collins R. UK Biobank: opportunities for cardiovascular research. Eur Heart J. 2019;40(14):1158-66.

2. Munafò MR, Tilling K, Taylor AE, Evans DM, Davey Smith G. Collider scope: when selection bias can substantially influence observed associations. International Journal of Epidemiology. 2018;47(1):226-35.

3. Haworth S, Mitchell R, Corbin L, Wade KH, Dudding T, Budu-Aggrey A, et al. Apparent latent structure within the UK Biobank sample has implications for epidemiological analysis. Nat Commun. 2019;10(1):333.

4. Sudlow C, Gallacher J, Allen N, Beral V, Burton P, Danesh J, et al. UK Biobank: An Open Access Resource for Identifying the Causes of a Wide Range of Complex Diseases of Middle and Old Age. PLoS Med. 2015;12(3):e1001779.

5. Bycroft C, Freeman C, Petkova D, Band G, Elliott LT, Sharp K, et al. The UK Biobank resource with deep phenotyping and genomic data. Nature. 2018;562(7726):203-9.

6. Mitchell R, Hemani G, Dudding T, Corbin L, Harrison S, Paternoster L. UK Biobank Genetic Data: MRC-IEU Quality Control, version 2 2019.

7. Willer CJ, Schmidt EM, Sengupta S, Peloso GM, Gustafsson S, Kanoni S, et al. Discovery and refinement of loci associated with lipid levels. Nat Genet. 2013;45(11):1274-83.

8. Kettunen J, Demirkan A, Würtz P, Draisma HH, Haller T, Rawal R, et al. Genome-wide study for circulating metabolites identifies 62 loci and reveals novel systemic effects of LPA. Nat Commun. 2016;7:11122.

9. Sinnott-Armstrong N, Tanigawa Y, Amar D, Mars N, Benner C, Aguirre M, et al. Genetics of 35 blood and urine biomarkers in the UK Biobank. Nature Genetics. 2021;53(2):185-94.

10. Voight BF, Peloso GM, Orho-Melander M, Frikke-Schmidt R, Barbalic M, Jensen MK, et al. Plasma HDL cholesterol and risk of myocardial infarction: a mendelian randomisation study. Lancet. 2012;380(9841):572-80.

11. Richardson TG, Sanderson E, Palmer TM, Ala-Korpela M, Ference BA, Davey Smith G, et al. Evaluating the relationship between circulating lipoprotein lipids and apolipoproteins with risk of coronary heart disease: A multivariable Mendelian randomisation analysis. PLOS Medicine. 2020;17(3):e1003062.
